# Mono- and Bi-Phasic Cellulose Acetate Micro-Vectors for Anti-Inflammatory Drug Delivery

**DOI:** 10.3390/pharmaceutics11020087

**Published:** 2019-02-18

**Authors:** Vincenzo Guarino, Rosaria Altobelli, Tania Caputo, Luigi Ambrosio, Sergio Caserta, Paola Calcagnile, Christian Demitri

**Affiliations:** 1 Institute for Polymers, Composites and Biomaterials, National Research Council of Italy, Naples 80125, Italy; rosaria.altobelli@gmail.com (R.A.); taniacaputo87@gmail.com (T.C.); ambrosio@unina.it (L.A.); 2 Department of Chemical, Materials and Production Engineering, University of Naples Federico II, Naples 80125, Italy; sergio.caserta@unina.it; 3 IMAST SCaRL, Naples 80133, Italy; calcagnile_paola@hotmail.it; 4 Department of Engineering for Innovation, University of Salento, Lecce 73100, Italy; christian.demitri@unisalento.it

**Keywords:** electrohydrodynamic atomization, core/shell structure, micro-carriers, drug release, oral delivery

## Abstract

In recent years, different processing technologies have been engineered to fabricate capsules or particles with peculiar properties (e.g., swelling, pH-sensitive response) at the micro and sub-micrometric size scale, to be used as carriers for controlled drug and molecular release. Herein, the development of cellulose acetate (CA) micro-carriers with mono- (MC) or bi-phasic (BC) composition is proposed, fabricated via electrohydrodynamic atomization (EHDA)—an electro-dropping technology able to micro-size polymer solution by the application of high voltage electrostatic forces. Image analysis allows identification of the process parameters to optimize morphology, in terms of size distribution and shape. Meanwhile, an accurate rheological study has enabled investigating the interface between CA solutions with different viscosities to optimize BC systems. Release tests have confirmed that BC carriers can retain the drug more efficiently in acidic conditions, also providing a more gradual and sustained release until six days, with respect to MC carriers. Hence, all these results have proven that biphasic architecture significantly improves the capability of CA microcarriers to release ketoprofen lysinate, thus suggesting a new route to design core/shell systems for the retarded oral administration of anti-inflammatory drugs.

## 1. Introduction

The fabrication of innovative devices with peculiar structural properties is raising a lot of interest with regard to the controlled release of bioactive molecules. Micro-sized capsules have been used in cancer therapy as depots to encapsulate anti-cancer agents [1], or more recently, as micro-scaffolds loaded with multiple agents to stimulate specific signaling pathways and instruct cellular responses in simulated biological micro-environments [2]. In all of these cases, an accurate design of polymer matrix properties is required to optimize the encapsulation of drugs or actives, to protect labile molecules from hazardous environmental conditions, and to define a tailored release profile as a function of the specific application. Accordingly, Dawes et al. have demonstrated that the release profile can be drastically influenced by the spatial distribution of molecules, strictly related to the size of microspheres [3]. Moreover, the effect of peculiar chemical and physical properties of carriers may be corroborated by the structural properties at the micro/sub-micrometric level, thus finely tuning the release profile by the control of the relative diffusion/degradation mechanisms [4,5].

In this context, core/shell structures able to confine drugs preferentially in a core region may allow fine control of the water/drug exchange via diffusion, as a function of the peculiar properties of the inner/outer phases, thereby influencing burst release extension [6]. Meanwhile, an accurate modification of the shell properties—i.e., thickness—may also contribute to define the active transport kinetics [7]. In the last decade, several technologies have been explored to design micro- and nano-structured platforms for biomedical use. Among them, electrohydrodynamic atomization (EHDA) identifies one of the most powerful bottom-up technologies rapidly emerging as promising and innovative tools for the development of micro/sub-micro-vectors for molecular and cell delivery [8]. The fundamental working principle is based on the capability to break up a complex fluid (i.e., viscous polymeric fluids, inorganic gels, or composite slurries) into charged droplets by means of electrostatic forces [9]. Through a careful optimization of process parameters and an appropriate definition of the atomization setup—depending on the peculiar properties of nozzle, needle, and collectors—complex active micro- and nanostructures may be variously engineered to face more relevant key challenges of healthcare (e.g., advanced chemotherapy, biomedical diagnostics, and tissue regeneration). In contrast with other atomization techniques (i.e., gas atomization [10], vacuum atomization [11], centrifugal atomization [12], rotating disk atomization [13], ultrasonic atomization [14]), EHDA has some relevant advantages, including relative ease of droplet generation, great control of droplet transport, ability to avoid coalescence of droplets due to an electric charge of the same polarity on the droplets, enhanced adhesion and deposition, and so on [15].

Herein, we propose the fabrication of mono-component devices made of cellulose acetate by a modified process configuration, based on the use of coaxial needles to design core/shell architectures able to confine anti-inflammatory drugs more efficiently for oral delivery applications.

## 2. Materials and Methods

### 2.1. Materials

Cellulose acetate powder (Mn 30 kDa, 39.8 wt % acetyl), Polyvynil alcohol (Mw 89–98 kDa), Tween 20, sodium hydroxide, acetic acid (C_2_H_4_O_2_), and acetone (C_3_H_6_O) were provided by Sigma Aldrich (Milan, Italy). Ketoprofen lysinate (KL) was kindly supplied by Dompè Pharmaceuticals (Milan, Italy).

### 2.2. Fabrication

**Figure 1:**
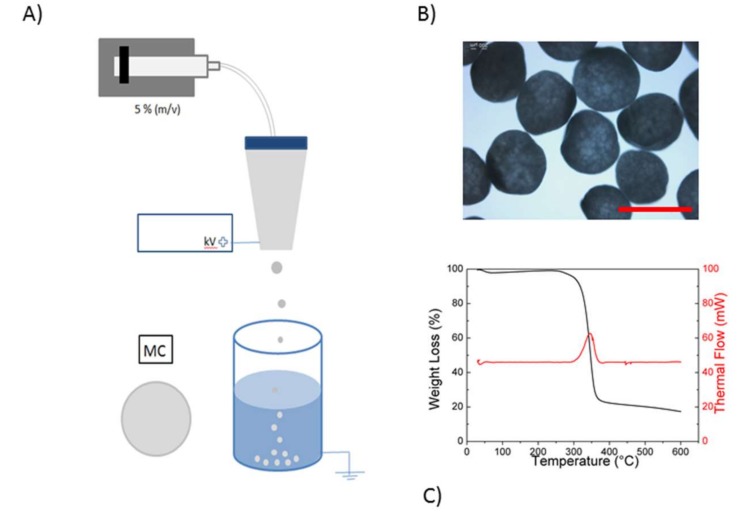
Ketoprofen lysinate (KL)-loaded mono-composition (MC) micro-vectors. (**A**) Scheme of capsule preparation; (**B**) qualitative evaluation of capsule morphology via optical microscopy (scale bar 500 µm); (**C**) thermal analyses: weight loss (in black) and thermal flow (in red) with respect to the temperature, measured by TGA and DSC instrumentation.

Cellulose acetate (CA) solution was prepared by dissolving 0.05 g/mL into an 80:20 (volume ratio) mixture of acetone and bi-distilled water. As for the drug-loaded samples, ketoprofen lysinate (KL) was added under magnetic stirring at room temperature up to obtain a clear solution. All the atomized particles—mono-composition (MC) and bi-phasic composition (BC)—were obtained by using a commercially available electrospinning setup (NF-500, MECC, Fukuoka, Japan) equipped with a tailor-made collector to form stable polymer droplets under controlled magnetic stirring (Figure 1). In the case of BC typology, two different CA solutions—5% *w*/*v* and 10% *w*/*v*—were respectively processed by a metal ultra-coaxial needle (MECC, Fukuoka, Japan)—inner channel 27G and outer channel 18 G—to form a core/shell system. Each polymer solution was placed in a 5 mL plastic syringe by connecting them to the spinneret. Once the pump system was working, MC or BC droplets were collected into an aqueous coagulation bath containing sodium dodecyl sulfate (SDS) at a concentration of 5 mM, and placed under magnetic stirring during the process in order to improve the surface stability of systems. Main process parameters were selected to optimize the final particle morphology: voltage ranging from 12 to 18 kV and a flow rate from 0.5 to 1.5 mL/h. All experiments were assessed by imposing a 15 cm gap—namely the distance between needles and ground collector—in a vertical configuration at 21–23 °C and 45–50% relative humidity. They were properly set to identify the best process condition to optimize the fabrication of MC and BC capsules loaded with ketoprofen lysinate (KL): *V* = 13 kV, *Q* = 2.5 mL/h for MC capsules; *V* = 13 kV, *Qint* = 0.5mL/h, *Qext* = 2.0 mL/h.

### 2.3. Morphological Analyses

The morphology of MC systems was preliminary investigated via optical scanning microscopy and scanning electron microscopy (SEM), (Zeiss-EVO40, Carl Zeiss Group, Oberkochen, Germany). All capsules processed by different conditions—i.e., under different values of the imposed flow rate and voltage—were compared. For each sample in suspension, some capsules were placed onto a microscope slide, and observed under transmitted light by a 2.5x-plan NEOFLUAR objective (Zeiss Axiovert 200, Carl Zeiss Group, Oberkochen, Germany). Meanwhile, for selected samples, SEM images were acquired in high-vacuum mode (20 KV), in order to investigate the cross-section and inner morphology of the systems. In all the cases, the morphological parameters were evaluated via image analysis.

### 2.4. Image Analysis

For the characterization of MC morphology, selected optical images were analysed for each typology. Different morphological parameters, including average diameter and distribution as well as shape factor, were calculated by the use of dedicated image analysis software (Image Pro Plus, ver. 7.0) onto a representative population of specimen (*n* > 80). For each particle, the maximum and minimum axis was measured. The circularity of the particle was estimated by calculating the parameter *D* = (*a* − *b*)/(*a* + *b*). Assuming an ellipsoidal shape, the volume and external surface was also calculated in agreement with previous studies [16].

### 2.5. Thermal Analyses

The thermal properties of MC and BC micro-vectors were also analyzed by thermogravimetric analysis/differential scanning calorimetry (TGA/DSC1 Star and System, Mettler Toledo, Zürich, Switzerland). All the tests were performed analyzing drug-loaded samples under a continuous and uniform nitrogen flow. An empty pan was used as a reference. For this purpose, the samples were heated from 25 °C up to 600 °C at 10 °C/min. The weight loss and the thermal flow were monitored during the test.

### 2.6. Rheological Analyses

Rheological investigation on cellulose acetate solutions used for the preparation of BC samples was assessed. Briefly, cellulose acetate solutions of 5% *w*/*v* and 10% *w*/*v* in a mixture of acetone/water (80/20 *v*/*v*) were stored in a sealed glass vial at room temperature. Different investigations were performed in order to fully characterize the solutions’ rheological properties; in particular, the trend of viscosity was investigated for both solutions, firstly by varying the shear rate in the range of 1–100 Hz, and then over time (2 min test) keeping the shear rate value constant at 1 Hz and 5 Hz, respectively. These tests were performed by means of an Ares rheometer (Rheometric Scientific Inc, Piscataway, NJ, USA), in a parallel plate configuration (25 mm plate diameter).

Viscoelastic properties like storage (G’) and loss (G”) moduli over frequency were measured. A strain sweep test was first performed, to select an appropriate strain amplitude at which the linear viscoelastic behaviour is observed. Measurements were then conducted in a constant strain (0.0025) mode as a function of frequency, from 0.1 to 100 rad/s. The results were recorded in terms of G’ and G’’ respectively. Each test was performed in triplicate, and the average was then calculated.

### 2.7. In Vitro Release

In vitro release tests were conducted onto KL-loaded systems in simulated biological fluids, simulating different pH conditions along the gastro-digestive tract. Hence, simulated gastric fluids (SGF) and intestinal fluids (SIF) were prepared according to the European guidelines of pharmacopoeia, in accordance with previous studies [17]. Samples were placed into 1 mL of medium and incubated at 37 °C under shaking. The released KL amount was evaluated at 260 nm using a UV-Vis spectrophotometer (Perkin-Elmer Victor X3, Milan, Italy). A quantity of 500 µL of the solution was replaced by fresh medium at each time step for measurements. All tests were conducted in triplicate for MC and BC sample populations. The encapsulation efficiency and drug loading were calculated as reported in previous studies [18,19].

## 3. Results and Discussion

In the last decade, several studies have been focused on the design of drug delivery systems to finely control release kinetics for a site-specific delivery, in order to reduce side effects and improve therapeutic efficacy and safety. In this work, new cellulose-based carriers were engineered via electrofluidodynamic techniques to achieve a sustained release of KL for several hours after the in vitro administration. In more detail, two different systems—MC and BC respectively—were fabricated via a mechanism of fluid breaking into micro-droplets driven by the application of electrostatic forces, recently defined as electro hydrodynamic atomization [20]. The term “electro” refers to the application of electrostatic forces to promote solution dropping, the term “hydrodynamic” refers to the fluid dynamic conditions applied to the viscous polymer solutions, and “atomization” refers to the capability of breaking a bulk liquid droplet into finely dispersed micro-droplets. This bottom-up technology is particularly suitable for synthesizing microstructures in the form of particles/capsules with different sizes and shapes, as a function of the capability of high-voltage electric forces to interact with and manipulate viscoelastic polymer solutions.

A conventional EHDA process for the fabrication of MC systems is schematized in Figure 1A. Under the application of electrostatic forces over the voltage threshold, the surface tension of CA solution was rapidly overcome, thus promoting the formation of a relatively monodispersed size distribution of droplets. Once collected into the SDS-rich water bath, particles with round-like shapes (Figure 1B) were forming, due to the ionic interactions of the CA solution with SDS and water into the coagulation bath. In particular, the diffusion of water into CA micro-droplets drastically reduced acetone concentration by a thermodynamically driven stripping mechanism, which promotes the fast precipitation of CA microcapsules. This phenomenon was also controlled by the local interaction between CA and SDS macromolecules, which concurred to stabilize particle shape during the acetone removal. Hence, CA microcapsules with a narrow size distribution were obtained (Figure 1B). It is noteworthy that no relevant modification of physical properties of CA capsules was recognized after the process, as confirmed by thermal analyses (Figure 1C): weight loss and thermal flow with respect to the temperature were comparable with those from the net CA, as reported elsewhere [21]. According to previous works, the first inflection point on the TGA signal (Figure 1C, black line) at temperature lower than 100 °C, was probably due to a weight loss (about 3%) caused by the evaporation of residual humidity. On the contrary, at around 360 °C, a substantial weight loss due to polymer degradation was noticeable. At the same temperature, on the DSC signal (Figure 1C, in red), the positive peak revealed the corresponding rise of the thermal flow due to the polymer degradation [22].

**Figure 2:**
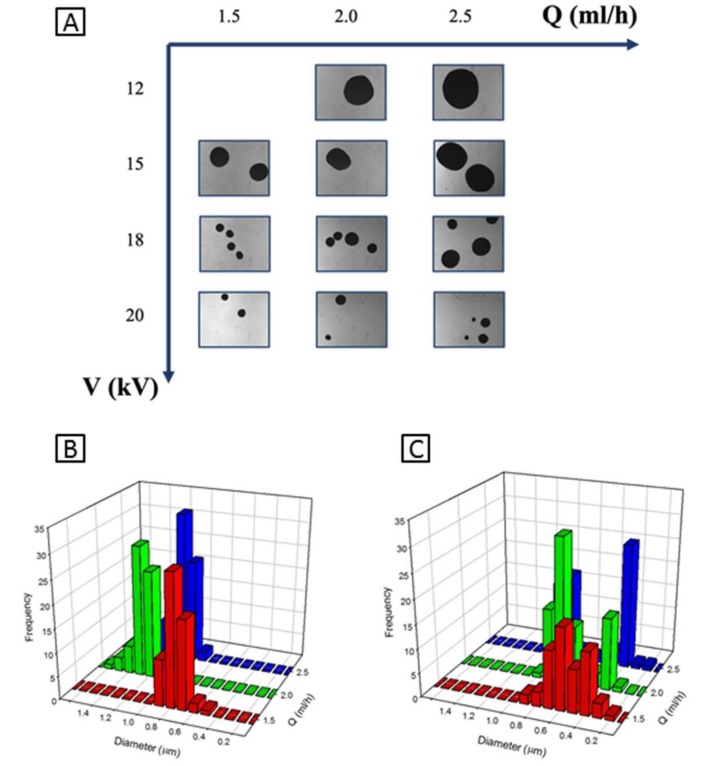
Optimization of electrohydrodynamic atomization (EHDA) for MC carriers: (**A**) qualitative evaluation of capsules morphology via optical microscopy and (**B**,**C**) quantitative evaluation of capsule diameters and size distribution calculated via image analysis. If the voltage is low enough, i.e., 15kV (**B**), then size distribution is uniform, while as the voltage increases, i.e., 18kV (**C**), then instability become predominant, causing heterogeneities in size.

In contrast, EHDA process conditions may drastically influence the morphology of MC capsules in terms of size and shape. In Figure 2A–C, a summary of the morphological characterization performed via optical microscopy supported by image analysis is reported to identify the best processing conditions (*V*, *Q*) to fabricate round-like capsules. Different sets of MC carriers were fabricated by singularly varying voltage from 12 to 21 kV, and flow rate from 1.5 to 2.5 mL/h.

The capsule shape, slightly elliptical, was initially approximated to a rounded sphere to measure the average diameter by image analysis software. The elaboration of optical images via dedicated software clearly shows a large variability in the average size, from 1248 ± 120 µm down to 287 ± 76 µm, underlining a strict size distribution in the case of lower applied voltage values (Figure 2B). As expected, as the flow rate increased, a significant increase in capsule size was recorded. However, by the application of higher voltage values from 18 kV, mainly predominant instability phenomena were recognized, with evidence of more remarkable heterogeneities in size causing the formation of two populations of capsules with different sizes (see the bi-modal size distribution measured in Figure 2C.) It is noteworthy that a slightly elliptical shape of CA capsules—less than 10%—was revealed by optical images; this effect was mainly due to the droplet elongation occurring into the coagulation bath, prior to the droplet stabilization. An accurate image elaboration was addressed to evaluate the effect of singular processing parameters on the singular capsule shape.

**Figure 3:**
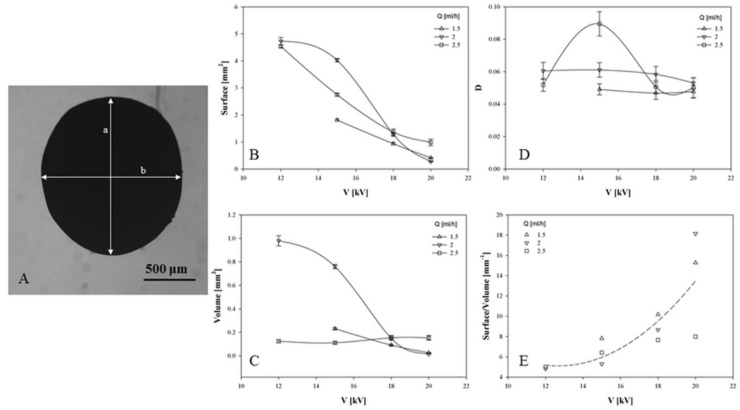
Detailed size and shape analysis of capsules. For each process conditions, both the axes of the ellipsoidal shaped capsules were measured (**A**). Surface (**B**) and volume (**C**) were calculated assuming an ellipsoidal shape. Particle ellipticity can be estimated by calculating the deformation parameter (**D**) and the surface/volume ratio (**E**). Each datapoint was calculated as the average of different measurements for about 80 samples, and standard error of the mean is reported as an error bar.

In particular, for each process condition—i.e., for each value of the imposed flow rate *Q* and tension *V*—a number of capsules ranging between 60 and 140 was analyzed, by measuring the mayor and minor axis (a and b in Figure 3A). Assuming each capsule was a rotational ellipsoid, symmetric respect to its major axis *a*, it was possible to calculate each capsule’s volume and external surface. In Figure 3B and Figure 3C, surface and volume are reported as a function of the voltage V, with parametric respect to the imposed flow rate *Q*. The average of the geometric quantity was measured over the entire sample population, and the standard error of the mean as an error bar was reported (see the lines indicated as guides for the eyes). As previously observed, capsule size is a decreasing function of the voltage, while in the range of parameters here investigated the influence of the flow rate on the size is not monotonic. The shape of CA capsules was also quantified. In Figure 3D, the deformation parameter *D* is reported as a function of the voltage, parametric in the flow rate. Finally, in Figure 3E the surface/volume ratio is also reported. A significant influence of the voltage V on this parameter was observed. The three data series, independently from the value of the imposed flow rate, have been fit to a polynomial curve, reported as a dashed line. This effect may be considered directly ascribable to the peculiar viscoelastic properties of the polymer solution, which is able to affect the droplet shape by the elastic deformation of the polymer solution at the tip of the needle, imparting an elliptical shape to the droplets’. In order to better control the shape, polymer solutions with different viscosities have been investigated in past literature [23]. Hence, two solutions with different viscosities have been used for the fabrication of biphasic CA capsules. This has allowed the containment of capsule elongation, also influencing specific functionalities for a controlled release of anti-inflammatory drugs. Recent studies have demonstrated the versatility and high feasibility of the EHDA process to particularize properties and functions of micro-sized devices by the easy customization of the process setup configuration, in order to obtain tailored systems for different applications in biomedical areas (i.e., regenerative medicine, drug delivery, bio-cosmetics, bio-packaging) [17,24] that are easily scalable for industrial production [25].

**Figure 4:**
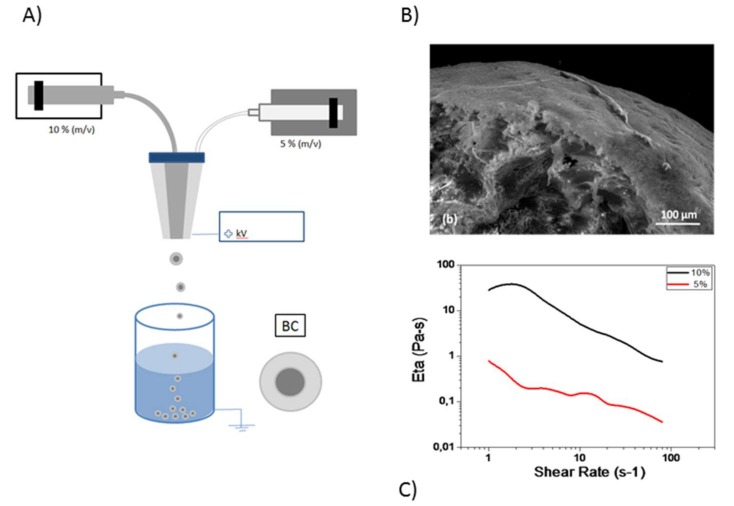
BC capsules: (**A**) scheme of preparation; (**B**) cross-section image via LV-SEM; (**C**) viscosity vs. shear rate in the case of inner (black) and outer (red) CA solutions.

In Figure 4A, the scheme of BC preparation was reported. In this case, the use of coaxial needles enabled simultaneously processing of two solutions with peculiar chemical/physical properties, thus imparting to the micro-carrier a core–shell structure [26], suitable for targeting and controlling the release of drugs and molecular species. Herein, two different CA solutions with different concentrations, 5% *w*/*v* (outlet) and 10% *w*/*v* (inlet) were used to generate microcapsules—i.e., only made of CA but with core shell architecture--to gradually release KL. SEM images (Figure 4B) show a zoom of a BC cross section, to qualitatively investigate its highly heterogeneous inner structure. In particular, a highly porous interconnected matrix was recognized at the outer regions. This is basically ascribable to the effect of local interactions occurring at the interface of the droplet, where a less concentrated CA solution promotes the diffusion of the surrounding coagulation medium and a gradual exchange of acetone at equilibrium. Meanwhile, a skin effect was also detected, probably due to the contribution of SDS concurrent to the surface stabilization. Notably, this peculiar morphology did not compromise the capability to entrap bioactive molecules. Indeed, the discontinuity in viscosity (Figure 4C) between the inner and the outer CA solution assures the creation of a stable interface that is better able to confine drugs into the inner and more viscous core of capsule, preventing massive and uncontrolled drug loss towards the external regions. As a consequence, the encapsulation efficiency of BC was about twice that of MC. An accurate investigation of the rheological properties was assessed onto CA solutions, in order to correlate the contribution of the relative viscosity to the final properties of MC and BC capsules in terms of drug release. The relationship between viscosity and shear rate was primarily evaluated, with shear rate varying in the range of 1–100 Hz. The results, shown in Figure 4C, clearly demonstrate a significant difference in viscosity, with typical shear-thinning behavior [27,28] independent from the solution concentration. At the beginning, the increment in the viscosity curve of outer CA solution was related to an initial inertia, which was mainly detectable in the case of higher concentrations. On the contrary, such an increment was not visible in the curve of the less concentrated solution, where signal noises tended to cover lower viscosity values. In the latter case, the values were closer to the lowest sensitivity of the transducer.

**Figure 5:**
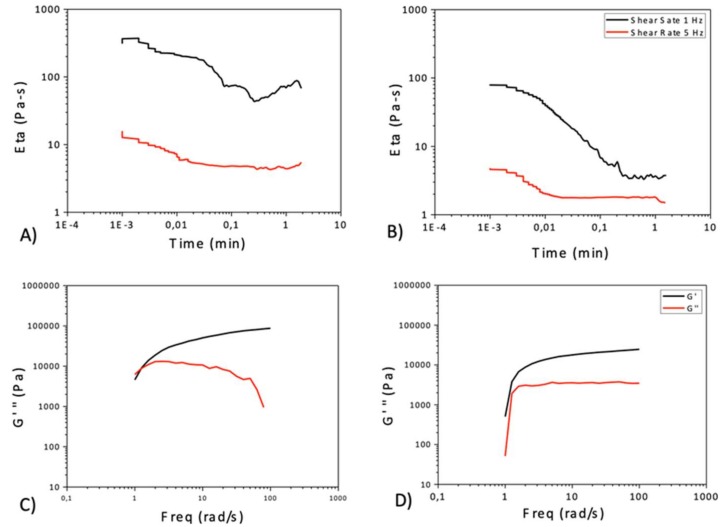
(**A**,**B**) Relationship between viscosity and time at different values of the applied shear rate (1 Hz and 5 Hz, in black and red, respectively), for the two cellulose acetate solution (10% (**A**) and 5% (**B**) concentrated). (**C**,**D**) Elastic module G’ (in black) and viscous module G’’ (in red) as a function of frequency, for both cellulose acetate solutions (10% (**C**) and 5% (**D**), respectively).

The rheological characterization was carrying out by analyzing the time-dependent viscosity of the two differently concentrated solutions at 1 and 5 Hz (Figure 5A,B). Two minutes was the chosen time test, in order to limit the effects of solvent evaporation. As demonstrated by Figure 4 and Figure 5A,B, the viscosity of the two solutions decreases by increasing both shear rate and time. As expected, the viscosity values in the case of more concentrated solution (Figure 5A) are always higher than the corresponding ones in the case of less concentrated solution (Figure 5B).

The characterization was finally addressed to the analysis of viscoelastic properties. Storage and loss moduli (G’ and G’’) were evaluated in the function of frequency by means of a frequency sweep test, with a strain value equal to 0.0025, falling into the linear range of both moduli. The results shown in Figure 5 indicate that in the case of 10% concentrated solution (c), at lower frequency values characterizing the initial part of the test, the storage (G’) and viscous (G’’) components of the solution are similar. Increasing the frequency makes G’ become clearly separated from G’’, showing the higher elastic response of the polymer. In that case, the elastic property of the solution is dominant, indicating that the formation of an organogel-like structure [29]. This behavior is not noticeable in the 5% concentrated solution (Figure 5D), probably due to the lower concentration of the polymer.

Hence, the morphological features of MC and BC capsules selected for the in vitro release tests are summarized (Table 1). The presence of a more viscous inner phase promotes an increase of particle size but a decrease of the deformation parameter (*D*), thus stabilizing the shape of capsules in the rounded form.

**Figure 6:**
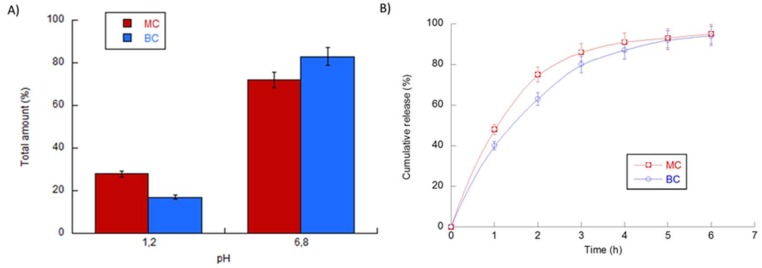
Release properties of BC and MC micro-vectors. (**A**) KL total amount (%). All samples were conditioned into simulated gastric fluids (SGF) (pH 1.2) for the first 2 h, and simulated intestinal fluids (SIF) (pH 6.8) for the next 6 h, until the end of release. (**B**) Cumulative release in SIF (%). All tests were conducted in triplicate for each time, for both sample populations.

KL in vitro release in simulated biological fluids of the gastro-intestinal tract was also investigated, in order to underline the main differences due to the presence of the core/shell architecture. Release profiles of MC and BC carriers are reported in Figure 6A,B.

At pH 1.2, a significant reduction of the percentage of the released drug was recorded, from 18% in the case of BC—to 26% in the case of MC. This result was strictly related to the higher capability of bi-phasic capsules with peculiar core/shell architecture. Indeed, the more viscous core of BC allowed a more efficient KL confinement with respect to MC, thus playing not only a storing role but also a protective function of inner bioactive molecules. Accordingly, at pH 6.8, a total amount of KL equal to 82% and 74% was recorded for BC and MC, respectively (Figure 6A). Moreover, a comparative evaluation of in vitro release profiles was also conducted in SIF (pH 6.8) until 6 h. Different KL release profiles were detected as a function of the peculiar architecture of the compared devices (Figure 6B). In the case of BC, with a core/shell structure, the fluid transport in the inner core, which is denser and more viscous then the outer shell, was partially hindered, and consequently, KL release was poorly delayed. In contrast, in the case of MC, with a homogeneous and less viscous body, the transport of KL was facilitated and KL was released more rapidly. During the first hours, a sustained release was reported, mainly driven by the presence of higher KL gradients. In particular, a significant discrepancy between BC and MC profiles was observed mainly until 3 h, with a gap of released KL of about 15–20% over the same time (Figure 6B). This peculiar release profile is promising for designing smart micro-vectors able to locally guide drug administration by the controlled swelling/adsorption properties of polymer matrices in response to microenvironmental stimuli, such as pH.

Hence, the use of core shell architecture in CA capsules, developed via EHDA, may be efficacious for controlling the release mechanism of drugs, if compared with other processing techniques (i.e., emulsion-solvent evaporation [30], the nanoprecipitation method [31], and the supercritical anti-solvent process [32]. Despite the total amount of drug that was released over the same time—from 6 to 8 h—the possibility of encapsulating the drugs into a more viscous solution, with respect to those conventionally used in other processes, allows the delivery of the active principle more slowly immediately after the administration, thus reducing the side effects due to the presence of a more pronounced burst release [33]. More interestingly, this mechanism could be also customized, to some extent, by appropriately varying the viscosity of the core solution, thereby giving the opportunity to design different BC systems with tailored release profiles for the selected administration of different drugs (i.e., anti-inflammatory, antibiotics, etc.) as a function of target demands.

## 4. Conclusions

In recent years, a fine control of chemical functionalities—that is, hydrophilic, partially hydrophilic, or hydrophobic groups like hydroxyl, carboxyl, amide, and sulfate—have allowed the fabrication of a large portfolio of chemically modified microparticles processed via conventional routes with different release properties. However, significant experimental evidence is increasingly remarking on the relevant impact of safety problems in the use of chemically modified devices for oral drug administration, mainly due to unexpected degradation and release of unsafe compounds rapidly adsorbed along the gastrointestinal tract. In this work, a novel technological approach, namely EHDA, has been investigated to design CA microparticles for safe oral delivery. Herein, we prove that modification of the KL release from CA micro-vectors may be directly induced by the peculiar biphasic architecture, without the use of chemical modification. A proper implementation of the EHDA process setup enabled us to design bi-phasic CA capsules with improved encapsulation and release properties, in comparison with mono-phasic ones, by imparting a core shell structure to the device. All the results in terms of release suggest a successful use of BC micro-vectors for the administration of KL or other anti-inflammatory drugs with hydrophilic behavior. Indeed, they firstly assure the minimization of side effects ascribable to the residence of drugs into the stomach, and secondly, to release drugs more efficiently (over 80%) along the small intestine mucosae, due to the peculiar pH-responsive behavior of CA, which is able to facilitate fluid transport at neutral/slightly basic micro-environmental conditions. A retarded release mechanism until 2 h after intake, followed by a sustained drug release over next 6 h, is very promising for developing versatile delivery systems for a sustained delivery of therapeutic agents in oral treatments.

## Figures and Tables

**Table 1:** Comparison of morphological parameters via image analysis for MC and BC capsules selected for in vitro release tests.

Morphological Parameters	MC	BC
Diameter [mm]	0.95 ± 0.01	1.71 ± 0.04
*D* [-]	0.09 ± 0.01	0.05 ± 0.01
Volume [mm^3^]	0.43 ± 0.01	2.52 ± 0.20
Surface [mm^2^]	2.75 ± 0.05	8.94 ± 0.49
